# Sonographic Diagnosis of COVID-19: A Review of Image Processing for Lung Ultrasound

**DOI:** 10.3389/fdata.2021.612561

**Published:** 2021-03-09

**Authors:** Conor McDermott, Maciej Łącki, Ben Sainsbury, Jessica Henry, Mihail Filippov, Carlos Rossa

**Affiliations:** ^1^ Faculty of Engineering and Applied Science, Ontario Tech University, Oshawa, ON, Canada; ^2^ Marion Surgical, Toronto, ON, Canada

**Keywords:** COVID-19, lung ultrasound, image processing, machine learning, diagnosis, segmentation, classification

## Abstract

The sustained increase in new cases of COVID-19 across the world and potential for subsequent outbreaks call for new tools to assist health professionals with early diagnosis and patient monitoring. Growing evidence around the world is showing that lung ultrasound examination can detect manifestations of COVID-19 infection. Ultrasound imaging has several characteristics that make it ideally suited for routine use: small hand-held systems can be contained inside a protective sheath, making it easier to disinfect than X-ray or computed tomography equipment; lung ultrasound allows triage of patients in long term care homes, tents or other areas outside of the hospital where other imaging modalities are not available; and it can determine lung involvement during the early phases of the disease and monitor affected patients at bedside on a daily basis. However, some challenges still remain with routine use of lung ultrasound. Namely, current examination practices and image interpretation are quite challenging, especially for unspecialized personnel. This paper reviews how lung ultrasound (LUS) imaging can be used for COVID-19 diagnosis and explores different image processing methods that have the potential to detect manifestations of COVID-19 in LUS images. Then, the paper reviews how general lung ultrasound examinations are performed before addressing how COVID-19 manifests itself in the images. This will provide the basis to study contemporary methods for both segmentation and classification of lung ultrasound images. The paper concludes with a discussion regarding practical considerations of lung ultrasound image processing use and draws parallels between different methods to allow researchers to decide which particular method may be best considering their needs. With the deficit of trained sonographers who are working to diagnose the thousands of people afflicted by COVID-19, a partially or totally automated lung ultrasound detection and diagnosis tool would be a major asset to fight the pandemic at the front lines.

## 1 Introduction

Severe acute respiratory syndrome coronavirus 2 is the third pathogenic human coronavirus to be identified with a predilection for causing severe pneumonia in 15–20% of infected individuals and 5–10% of all cases requiring critical care. First emerged in Wuhan, China, it has quickly spread across the world Buonsenso et al. (2020). Severe forms of the infection are commonly characterized by pneumonia, lymphopenia, exhausted lymphocytes, and a cytokine release syndrome. As the COVID-19 epidemic develops, there is a strong desire for fast and accurate methods to assist in diagnosis and decision making Huang et al. (2020), Born et al. (2020a). The outward symptoms are similar to that of influenza and thus laboratory testing is required for diagnosis. The most common techniques that have been employed include ribonucleic acid analysis from sputum or nasopharyngeal swab alongside chest radiographs. However, these tests are not always able to detect this disease.

COVID-19 preparedness and response critically rely upon rapid diagnosis and contact tracking to prevent further spread of the infection. With a surge in new cases, particularly those requiring critical care, monitoring the disease can help healthcare professionals make important management decisions. While CT is a proven tool for diagnosing COVID-19, it has limitations that make routine use impractical: CT is not widely available, turnaround times are long, and it requires patients to be moved outside of their unit Hope et al. (2020) and reported sensitivities vary, as per Hope et al. (2020). Safely using CT machines during the pandemic is logistically challenging and can overwhelm available resources. Even with proper cleaning protocols, CT scanners could become a source of infection to other patients who require imaging.

Amidst the rush to use CT scans and develop image processing algorithms to detect COVID-19 in CT images, researchers seem to have given little attention to a much more convenient and simpler imaging method: Lung ultrasound (LUS), Buonsenso et al. (2020). LUS has been used for decades for diagnoses and patient monitoring in a variety of respiratory diseases including pneumonia and acute respiratory distress syndrome, as per Staub et al. (2018) and Lichtenstein (2009). Very recently, it has been proven to also have the ability to detect manifestations of COVID-19 in the images when the examination is performed accurately as shown by Huang et al. (2020), Thomas et al. (2020), and Buonsenso et al. (2020). LUS has many appealing features that make its application to COVID-19 diagnosis and monitoring quite advantageous. It uses basic technology available at a much larger volume than CT scans and is free of ionizing radiation. It is also non-invasive, repeatable, cost-effective, and unlike CT-scan, LUS can be performed at a patient’s bedside. Furthermore, the issue of viral cross-contamination with LUS machines is nearly nonexistent. Sterilizing ultrasonography equipment is quite easy and is currently done hundreds if not thousands of times per week in a single hospital. More subtly, thanks to the prompt availability of LUS, patients may benefit from a lower threshold for performing LUS examination than what is required for CT tests. Thus, earlier and more frequent lung examinations can be offered, even in COVID-19 assessment centers outside of hospitals. Furthermore, infected but discharged patients could be evaluated with lung imaging directly in their homes. This is particularly important with respect to long-term care homes and in regions experiencing a deficit of available hospital beds.

With the completion of a reliable diagnostic algorithm and handheld tool, it will be possible to diagnose patients where there is an absence or limited number of practitioners, such as in rural and isolated communities. This can assist in better managing medical resources by providing a quick and reliable way to triage patients.

Early diagnosis allows for timely infection prevention and control measures. Patients with mild disease do not require hospitalization, unless there is concern for rapid deterioration. Thus, in the short term, a more systematic way to help healthcare professionals identify cases and assess the risk of progression to severe or critical conditions, or from acute to subacute conditions, can help better manage scarce resources in hospitals. Thus, routine use of LUS can help the fight against COVID-19 in several ways:

- LUS offers a supplementary screening tool available in any healthcare center. It can allow for a first screening to discriminate between low and high-risk patients. Routine LUS is much easier to implement as a screening tool than other imaging methods and thus earlier and more frequent lung examinations can be offered, even directly in COVID-19 assessment centers outside of hospitals.
- In the absence of sufficient COVID-19 testing kit availability, LUS can assist in diagnosing patients;
- LUS images can be obtained directly at bedside reducing the number of health workers potentially exposed to the patient. Currently, the use of chest X-Ray or CT scan requires the patient to be moved to the radiology unit, potentially exposing several people to the virus. With LUS, the same clinician can visit the patient and perform all required tests. This is a primary point since recent data shows that in severely affected countries about 3–10% of infected patients are health workers, worsening the serious problem of health professionals’ shortage Buonsenso et al. (2020);
- Discharged patients can be actively monitored with LUS imaging directly in their homes. This is crucial in long-term care homes and in regions with saturation of admission in hospital beds;
- Portable ultrasound machines are easier to sterilize due to smaller surface areas than CT scans;
- LUS is radiation free and can be performed every 12–24 h, allowing close monitoring of clinical conditions and also detecting very early change in lung involvement;
- LUS can be easily performed in the outpatient setting by general practitioners. This would also allow a better pre-triage to determine which patients should be sent to a hospital;
- Lastly, LUS is an inexpensive instrument and can be easily deployed in resource-deprived settings. In case of a massive spread, traditional imaging such as CT scan is much more difficult to be performed compared to LUS.

A database of LUS ultrasound images is being collected by researchers worldwide Born et al. (2020a), Roy et al. (2020). Reports issued from this data have identified common structures seen in LUS on patients with confirmed cases of COVID-19. The data has revealed trends in LUS images that provide markers for the disease. However, these indicators have also been seen in other respiratory infections, but COVID-19 has some unique distinguishing features. Some of these investigations have drawn from limited datasets: 1 case in Thomas et al. (2020) and 20 in Huang et al. (2020), to over 60,000 images in Soldati et al. (2020). Although there is strong evidence that LUS can diagnose and monitor COVID-19, it is important to acknowledge that there is a spectrum of clinical manifestations of the virus in LUS images during the clinical course of the infection. Even though image-based patterns are intuitively recognisable, they may be mistaken with manifestations of other respiratory diseases. Furthermore, according to the standardized protocol for point-of-care LUS and grading score system proposed in Italy by Soldati et al. (2020), a lung examination requires multiple LUS scans obtained at different locations on the chest. It becomes hard to reconstruct a mental map of a required set of up to 14 scans, and image quality and interpretation are largely operator-dependent. These issues suggest that LUS diagnosis would benefit from a standardized approach, common language, and uniform training, which may not be feasible in the time of pandemic. Thus, there is an urgent need to develop computer-aided methods to assist with sonographic diagnosis of COVID-19.

This paper provides a review of contemporary methods for both the segmentation and classification of LUS and is organized as follows: The next section provides a review of existing manual diagnostic techniques currently being employed around the world. Section 3 delivers a narrative on proven techniques on LUS image segmentation found in literature. Section 4 does the same but with classification. Lastly, the paper discusses parallels between different methods and allow readers to decide which particular method may be best for their needs. With the deficit of trained sonographers who are working to diagnose the thousands of people afflicted by COVID-19, a partially or totally automated LUS detection and diagnosis tool can have a tremendous impact in the battle against COVID-19. Let us start with narrative on how conventionally COVID-19 and other lung diseases are examined and diagnosed using LUS. It is important to note, however, that not all of the methods presented in this paper have been specifically used as a diagnostic tool for COVID-19, but they have the potential to be used as such. As COVID-19 is a new virus, little work has been done to develop detection tools. This paper is meant to act as a guide for methods that have been proven to diagnose pneumonia and other respiratory pathologys indicative of COVID-19.

## 2 The Bases of Lung Ultrasound Diagnosis of COVID-19

Since the end goal is to at least partially automate the process of LUS diagnosis, an understanding of how LUS images are acquired is necessary.

### 2.1 LUS Examination Protocol

Before moving any further, it is important to outline the basic principles of LUS and how it is being applied to COVID-19. LUS images offer real-time insight into the state of eration of the lung, i.e., the air to fluid ratio in the lung, which distinguishes normal eration from respiratory illnesses.

**FIGURE 1:**
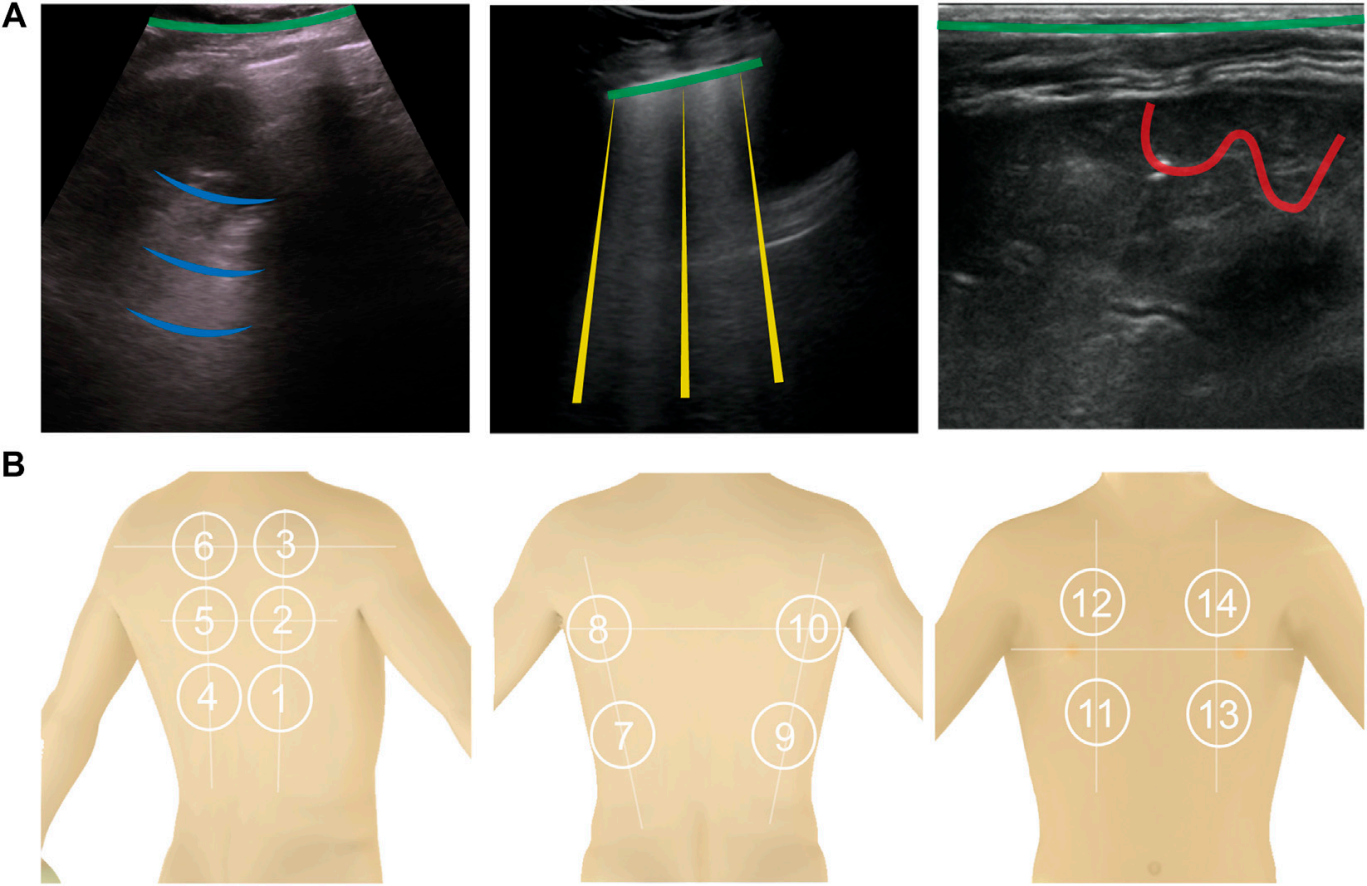
**(A)**: Shows the four types of lines found in LUS images. A-lines are shown in blue, B-lines are yellow, C-lines red, and the pleural line is green. **(B)**: 14 anatomical scanning locations for LUS diagnosis. From left to right are scanning locations on the back [with the vertical paravertebral line, spine of shoulder blade (upper horizontal line) and interior angle of shoulder blade (lower horizontal line)], sides (showing the mid-axillary lines on the left and right sides and internipple line), and front of a torso (showing the internipple line).

Normally erated lung: Since ultrasonic energy is rapidly dissipated in the air, in a normally erated lung the only detectable structure is the pleura, observed as a hyperechoic horizontal line (see Figure 1, green lines). The pleural line moves synchronously with respiration—this is called lung sliding. In addition, successive hyperechoic horizontal lines appear below the pleural: the A-lines (blue). These artifacts along with lung sliding represent a sign of normal content of air in the lung by Gargani and Volpicelli (2014). See Figure 1A.

Interstitial lung disease: When the state of eration decreases due to the accumulation of fluid or cells, the ultrasound beam travels deeper in the lung. This phenomenon creates vertical reverberation lines known as B-lines (comet-tail artifacts outlined by yellow lines in Figure 1A). Hyperechoic B-lines start at the pleural line, extend to the bottom of the image without fading, and move with lung sliding. The lower the air content in the lung, the more B-lines are visible in the image. Multiple B-lines in certain regions indicate lung interstitial syndrome.

Lung consolidation: When the air content further decreases to the point of absence of air, with some abuse of terminology, the lung becomes a continuous medium where ultrasound waves cannot reverberate. The LUS image appears as a solid parenchyma, like the liver or the spleen. Consolidation is the result of an infectious process, a pulmonary embolism, obstructive atelectasis, or a contusion in thoracic trauma. Additional sonographic signs are needed to determine the cause of the consolidation in order to attribute it to COVID-19 such as the quality of the deep margins or the presence of air or fluid bronchogram Huang et al. (2020). In Figure 1A, consolidation is indicated by the presence of the C-lines highlighted in red.

The recommended acquisition protocol for COVID-19, as proposed by Soldati et al. (2020), screening includes 14 intercostal scans in 3 posterior, 2 lateral, and 2 anterior areas, currently considered “hot areas” for COVID-19 (Figure 1B). Each scan is 10 s long so that lung sliding can be visualized. For patients who are not able to maintain the sitting position the echographic assessment may start from landmark number 7, as per Soldati et al. (2020). Once the images are acquired, each scan is analyzed and classified following the 3-point score summarized below Huang et al. (2020). Practically, the device to do this would need to be robust, cheap, and easily cleanable. The software would need to be able to be used by non-professional sonographers (i.e., nurses, etc.).

Specific manifestations of COVID-19 include:

- COVID-19 foci are mainly observed in the posterior fields in both lungs, especially in the posterior lower fields;
- Fused B lines and waterfall signs are visible under the pleura. The B lines are in fixed position;
- The pleural line is unsmooth, discontinuous and interrupted;
- The subpleural lesions show patchy, strip, and nodule consolidation;
- Air bronchogram sign or air bronchiologram sign can be seen in the consolidation; and
- The involved interstitial tissues have localized thickening and edema, and there is localized pleural effusion around the lesions;

### 2.2 Diagnosis

After the images are taken from the 14 intercostal positions, they can be analyzed to determine the presence of COVID-19 pneumonia. Depending on the results from the sonographer, a score is assigned to the LUS images to indicate the severity of the disease present, if any. The score is from 0 to 3, 0 indicating a healthy lung and 3 indicating a heavily diseased lung.

- Score 0: The pleural line is continuous and A-lines are present indicating a normally erated lung;
- Score 1: The pleural line is indented and below the indent B-lines are visible. These are due to the replacement of volumes previously occupied by air in favor of intercostal tissue;
- Score 2: The pleural line is severely broken and consolidated areas appear below the breaking point (C-lines and darker areas). The C-lines signal the loss of eration and the transition;
- Score 3: The scanned area shows dense and largely extended white lung with or without C-lines. At the end of the procedure, the clinician classifies each area according to the highest score obtained. Huang et al. (2020) further suggests that COVID-19 has other specific manifestations in LUS, mainly observed in the posterior area: Fused B-lines; the pleural line is unsmooth, discontinuous or interrupted; and the subpleural lesions show patchy, strip, and nodule consolidation in which air bronchogram can be seen. The interstitial tissues show obvious thickening and edema, the pleura shows localized thickening, and there is localized pleural effusion around the lesions.

**FIGURE 2:**
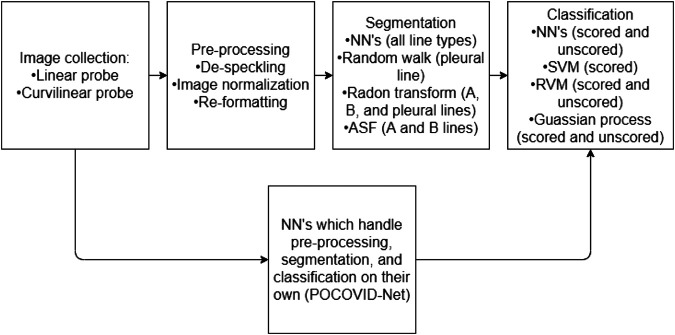
A flow chart representing a normal work flow for LUS images processing. The flowchart has two parallel components, illustrating that typically the stages of image collection, processing, segmentation, and classifications are performed in a linear fashion. However, the parallel component is to illustrate that some neural network methods can be trained in order to handle the entire process (from collection to classification) as one black-box solution.

There are numerous methods, presented in the following sections, which give medical researchers the tools required to pre-process, segment, and classify LUS images (Figure 2).

## 3 Segmentation of COVID-19 Manifestations in LUS

Segmentation divides an LUS image into smaller classifiable sections. This means identifying the pleuralline and the presence of A-lines, B-lines, or consolidated regions of the image. Thus, segmentation plays the role of interpreting what manifestations are held within the LUS image. Pre-processing is necessary as raw LUS images can be noisy and difficult to interpret.

### 3.1 Image Pre-Processing

Ultrasound images are noisy, often lack contrast, and contain artifacts such as attenuation speckles, shadows, and signal dropouts [Noble and Boukerroui (2006)], making image segmentation a difficult task. Furthermore, images collected using an ultrasound machine will differ between different models and types of probes. Pre-processing is almost always done on LUS images to enhance their quality and prepare them for further processing. One of the most common pre-processing operations is binarization. It converts pixels in a gray scale image into a black and white image (with pixels either on or off) based on the intensity of the pixel and a threshold value Correa et al. (2018). The choice of the threshold value changes the features that will be visible in the processed image.

Image normalization is required to offset any scaling differences between different images caused by gain adjustments on the ultrasound device. In case the gain settings are not known, Brattain et al. (2013) propose the use of the image peak approach for enhancing the image which minimizes the potential for differences in gain during the recording process from affecting the algorithm. Image reformatting may be necessary depending on the method used. In Cristiana et al. (2020), all images were reformatted to be in a consistent rectilinear format so no matter what transducer was used in order to take the images, they could be processed in the same way. Brattain et al. (2013) performs a similar operation where each frame in a video was reformatted and normalized so that difference in gain setting during the original recording was reduced. This further minimizes discrepancies between data sets.

### 3.2 Pleural Line Detection

The first set of segmentation methods focuses on the detection of the pleural. This first step is typically to exclude the area above the pleural (e.g.,: noise from the rib bones) from segmentation as it has no diagnostic importance aside from acting as a reference point.

The method presented in Moshavegh et al. (2016) and Moshavegh et al. (2018) employs the random walk technique to automatically detect the pleural line. A classical random walk algorithm, introduced by Grady (2006), is a method for image segmentation that can be either interactive or automatic. In this method a set of pixels called seeds is selected and given a label. Random walkers are then used to identify regions containing the labeled seeds. The method was adapted to ultrasound imaging in Karamalis et al. (2012). Since the ultrasound images contains artifacts and noise, the walkers are constrained using a confidence map constructed based on the image quality. This simple method is easy to implement as it uses a well-known image segmentation technique. One notable consideration is that a starting point must be chosen carefully Moshavegh et al. (2016), Moshavegh et al. (2018). Additionally, it remains unknown if this method is suitable for identifying the pleuralline in patients with score greater than 2, as in severe cases where the pleuralline can be discontinuous.

In Moshavegh et al. (2016) random walk is combined with alternate sequential filtering to detect the presence of pleurallines. A similar approach was proposed by Carrer et al. (2020) but instead of random walk, the method uses Hidden Markov Model (HMM) and Viterbi Algorithm (VA). It can detect discontinuous pleural lines, which is a direct advantage over the random walk method. Based on experimental evaluation, the algorithm can detect the pleuralline in a heterogeneous data set collected from various sources. Another method proposed in Correa et al. (2018) finds the pleuralline using in two steps. First, the image is binarized and divided into narrow vertical slices. Each slice is then divided in half by a line, which is moved such that the number of *on* pixels is equal above and below it. A curve is then fitted along to the points on each of the lines such giving the approximate location of the pleuralline.

One of the most common methods for line detection is the Radon transform, which projects the density of an object in an angular coordinate system Anantrasirichai et al. (2016), Anantrasirichai et al. (2017). The Radon transform can be used to identify the pleuralline by searching for the brightest horizontal line which they define as one with the $90^{\circ }\pm 20^{\circ }$. An improved version of this method presented in Karakus et al. (2020) was tested in LUS images of COVID-19 patients. One of the main advantages of this method is its simplicity. The pleuralline can be easily identified as it is always the brightest object in the image. As a major shortcoming, detectable lines are straight, meaning that this method can only approximate the actual position of the pleuralline and is more suitable for detecting A and B lines.

### 3.3 A and B Line Detection

Finding pleurallines is an important aspect of LUS imaging, however often times medical professionals are more interested in locating and segmenting the A and B lines that are characteristic of healthy and unhealthy lung conditions. This section describes some of these methods.

In a Radon transform A-lines can be identified as horizontal lines, ones with an angle $90^{\circ }\pm 20^{\circ }$, with a lower brightness than that of the pleuralline. Similarly, B-lines can be identified by searching for horizontal lines with angle $0^{\circ }\pm 20^{\circ }$. Anantrasirichai et al. (2016) and Anantrasirichai et al. (2017) proved that using such a method can indeed differentiate between these different lines.

Brattain et al. (2013) presented one of the first methods used to identify B-lines in LUS. The method converts the conic ultrasound image into a rectangle and divides it into columns. The B-lines are identified by finding columns through analyzing the brightness profile of each column, and searching for columns with a high, uniform intensity, spanning the length of the column. This method, though simple, requires the detection parameters to be tuned depending on the model of the ultrasound machine and the probe used to detect images. This method was later improved upon by applying a series of morphological operations and filters to the image to more accurately segment B-lines. Moshavegh et al. (2016), Moshavegh et al. (2018), and Brusasco et al. (2019) all used alternate sequential filtering^1^ (ASF) with an axial-line structuring element to consolidate regions containing disjointed elements of the B-lines into continuous vertical shapes. Moshavegh et al. (2016) applied the Top-Hat filter to distinguish between connected B-lines, while Brusasco et al. (2019) scans the image laterally in search of long columns that contain mostly bright pixels. The location of the B-Lines can be adjusted using Gaussian model fitting method, as shown in Moshavegh et al. (2018).

A similar approach in Correa et al. (2018) has been shown to aid in the identification of A-lines. Like in the previous methods, only the region below the pleuralline is considered. A procedure named *close method* is applied to the image to emphasize the shape of regions possibly containing A-lines. A-lines are identified by adding the brightness values of each row.

### 3.4 C-Lines (Consolidations)

There presently are no methods provided in literature specifically tailored for segmenting lung consolidations for LUS. However, there is potential for doing so. In Nazerian et al. (2015), a method for manually imaging pneumonia consolidations in LUS was presented. In this paper, recommendations for what to look for are included. The consolidations due to pneumonia usually contain dynamic echogenic structures that move with breathing. They may also contain multiple hyperechogenic spots, due to air trapped in the small airways, with associated focal B-lines. This is typically characterized by a large dark spot in the LUS, caused by pleura breakdown, as shown by Volpicelli et al. (2010). Lung consolidations are superficial and relatively easy to spot by lung ultrasound, as per Lichtenstein (2015). The methods presented by Correa et al. (2018) or Brattain et al. (2013) could potentially be applied to properly segment LUS images with lung consolidations present. A tool for identifying lung consolidations is important as C-lines are required for LUS diagnoses.

### 3.5 Neural Networks Based Segmentation

A more modern approach to segmenting LUS images involves using neural networks and deep learning. Convolutional Neural networks (CNNs) are a type of neural network (NN) specifically designed for processing, identifying, and detecting features in images or sounds tracks. These networks use deep learning methods and require hundreds or thousands of images with features labeled. To this end, large datasets are required for training and testing of CNNs. To this date, there are mainly two datasets of LUS images of COVID-19 patients. First, Roy et al. (2020) presented the Italian COVID-19 LUS DataBase (ICLUS-DB) composed of 277 LUS videos from 35 patients, 17 of whom were diagnosed with COVID-19, four were suspected, and 14 were healthy, with a total of 58,924 frames. Each image was labeled using the scoring system proposed by Soldati et al. (2020), seen earlier in Section 2.2. Second, the lung point-of-care ultrasound (POCUS), Born et al. (2020b) dataset Born et al. (2020a) contains 39 videos of COVID-19 patients, 14 videos of patients with bacterial pneumonia, and 11 healthy individuals for a total of 64 videos and 1,103 images. Both data sets were collected using a variety of ultrasound scanners and probes by sonographers in multiple different hospitals. The dataset includes ultrasound images of patients with bacterial pneumonia which is an important distinction when attempting to diagnose a patient with COVID-19. On the other hand, ICLUS-DB does only consider COVID-19 patients, but the data is labeled with the severity of the infection.

CNNs have been used previously to detect B-lines in patients with pneumonia. One weakly-supervized network built to detect B-lines in real-time was proposed by van Sloun and Demi (2019). The CNN uses 12 convolutional layers and incorporates a gradient-weight class-activation mapping (grad-CAM) which identifies the regions where B-lines are located. More importantly, the network learns how to identify B-lines based on data labels that only indicate if B-lines are present. Since the network does not need a labeled dataset for training, it is easy to implement and use. Note, however, that this network is not able to count the number of B-lines in an image, though the number of lines is an indication of the state of eration of the lung. Due to layer pooling, the output map highlighting the regions containing B-lines has low resolution. Wang et al. (2019) presents a four layer, semi-supervized CNN capable of measuring the number of B-lines in the images. The network was trained on dataset labeled with only the number of visible B-lines without specifying their location. The network can count the number of B-lines, but it is not able to identify their location. A similar approach is used to analyze brightness profiles from LUS data with artificial neural networks (ANN) in Barrientos et al. (2016), Correa et al. (2018).

Another CNN-based method that does not share the limitations described earlier has been proposed by Kulhare et al. (2018), who uses a Single Shot Detector (SSD) to identify the locations of the pleural, A, B, and C-lines. SSDs, introduced by Liu et al. (2016), are a fast and accurate method used to identify objects in pictures. The method uses feature maps generated by a 16 layer CNN presented in Simonyan and Zisserman (2014) to fit bounding boxes around the features. The network training requires training data with ultrasound images with target features locations fully annotated. This supervised algorithm has a sensitivity of 85% on animal specimens but cannot be used for COVID-19 until the two available datasets are annotated.

In contrast, the approach presented by Roy et al. (2020) uses a CNN with a Spatial Transform Network proposed by Jaderberg et al. (2015). It applies linear transformations to the feature maps of the image allowing features to be identified in any orientation. This enables the network to identify the regions of interest by itself. As a result, the network can provide feature localization without great level of supervision. Based on experimental validation, Roy et al. (2020) claim that this approach outperforms the one proposed in van Sloun and Demi (2019).

## 4 Image Classification

After LUS images have been segmented, they must be classified to provide diagnosis. Using the presence of B-lines, consolidation, etc., a classifier can assign a label to the previously segmented images which can then be used as a basis for diagnosis and prognosis Correa et al. (2018). There are two main methods of classifying LUS images: 1) Feature-based classification where segmented features are analyzed stochastically, and 2) learning-based methods such as NN’s which act more as a black box solution. They are trained to classify images based on geometric patterns that are present in certain diseases in the LUS images. This section discusses some available methods for segmented LUS image classification.

### 4.1 Neural Network Classification

There are mainly two NN methods used to classify images, firstly using pre-segmented images, where regions of interest are segmented by an expert and then fed into a NN, or secondly, a NN may be trained to do both segmentation and classification. Some of the networks discussed in Section 3.5 are CNN’s focused on finding and segmenting features in LUS images.

An interesting manipulation of data is the brightness profile of vectors method presented by Correa et al. (2018). In this method, LUS images of healthy lungs and with pneumonia are distinguished from one another by the brightness profile of the raw LUS data. The brightness profile being the profile that represents a single vector of ultrasound data as strong reflected ultrasound waves are interpreted as “bright”. The brightness profile of healthy lung tissue is characterized by smooth, exponentially decaying brightness, whereas unhealthy lung tissue has erratic brightness and non-exponential decay. Rib bones have an abrupt drop on brightness right below the pleuralline.

Cristiana et al. (2020) proposed a direct improvement to Correa et al. (2018) where a secondary NN was trained using softmax activation as a multiclass classifier. The method classifies whether B-lines are present and the multiclass classification network scores the images based on the scoring system presented by Soldati et al. (2020). The two models, binary and multiclass, were trained separate from one another. The binary classifier had a sensitivity of 93% and a specificity of 96%^2^ as compared to a medical expert classifying the same images. Agreement between the multiclass severity scoring system and a medical expert was 93%±1.

Similarly, Born et al. (2020a) presents POCOVID-Net, the first CNN for identifying COVID-19 through LUS, which uses VGG-16, as established CNN, pre-trained on ImageNet (Krizhevsky et al., 2012) for image feature extraction. It uses a pre-trained 16 layer CNN from Simonyan and Zisserman (2014) to extract lower level features such as textures and shapes. The last three layers of the network were further trained using POCUS dataset to differentiate between patients who were diagnosed with COVID-19, bacterial pneumonia, and healthy individuals. The network uses softmax activation to classify images and had an overall accuracy of 89%. It’s sensitivity and specificity for detecting COVID-19 in particular was 96 and 79%. van Sloun and Demi (2019) outline a method for CNN’s to segment and classify LUS images for B-lines. This method is one of the few which is capable of real time classification by exploiting GPU acceleration. It had an *in-vitro* accuracy of 91.7%, and an *in-vivo* accuracy of 83.9 when using the ULA-Op transducer, a research platform, and 89.2% using a Toshiba transducer. The network, with six layers, used softmax activation, just as Correa et al. (2018) and Cristiana et al. (2020). The greater accuracy in *in-vitro* data was due to analyzing *in-vitro* images, while the *in-vivo* data were videos as the videos are more complex and variable than the images to analyze as even the breathing of a patient is enough to make B-lines more difficult to detect. Further the presence of intercostal tissue, not present in the *in-vitro* data further complicates its processing. Therefore, a loss of resolution and classification accuracy is expected. van Sloun and Demi (2019) used imagenet a popular CNN architecture, as a basis for their pre-trained neural network, therein easier to train to perform particular tasks.

In Kulhare et al. (2018) a binary classifier, indicating presence or lack thereof, based off the Inception V3 SSD Convolutional Neural Network architecture. The system was trained to classify LUS images with A, B, and pleural lines as well as lung tissue consolidation. Overall, its pleuralline classification was 89% accurate.

Despite the suitability of NN’s for LUS image classification, they are often computationally heavier and require greater training sets than other methods. Stochastic methods provide a lighter option which are just as accurate which may be better suited for a portable LUS device.

### 4.2 Stochastic Classification

Stochastic classifiers are purpose-built classifiers which use statistical regression and image filtering to analyze the segmented images which are fed to them and then classify the image contents.

Brusasco et al. (2019) proposed an off-line method to segment and classify the quantity of B-lines, similar to the CNN model proposed by Wang et al. (2019), in LUS images. The end goal was to create an automated method of determining extravascular lung water. The algorithm scored the segmented gray-scale LUS images. B-lines are classified when the filtered images are scanned and white pixels are measured to make up ¿50% of the total vertical length of the image. Using statistical regression on the segmented LUS images, the total number of B-lines present can be quantified. However, classifying images when many B-lines are present is difficult as they coalesce and are imaged as singular B-lines as opposed to multiple, close-by B-lines.

In Carrer et al. (2020) a support vector machine (SVM) classifies and scores pleural lines. The SVM is fed segmented partitioned United States images whose features were fed into Gaussian radial basis function kernel, a type of SVM classifier known to have a better convergence time than polynomial kernels. The SVM classifier was chosen over an NN as it requires significantly less data to train, which is pertinent as COVID-19 training data is presently lacking. The classifier was applied to linear United States probe and convex United States probe data separately, and the accuracy for the linear and convex probes were 94 and 88%.

A similar method described by Veeramani and Muthusamy (2016) is to use two Relevance Vector Machines, a Bayesian framework for achieving the sparse linear model as per Babaeean et al. (2008), to classify the LUS images as healthy or unhealthy, and if unhealthy what disease is present. RVM’s provide a probabilistic diagnosis, as opposed to the discrete diagnose obtained with SVM’s. The method offered better accuracy, sensitivity, and specificity than SVM and NN methods. While first RVM classifier was a binary, the second RVM classifier was a multiclass classifier capable of noting which diseases are present in the lung including: respiratory distress syndrome, transient tachypnea of the newborn, meconium aspiration syndrome, pneumothorax, bronchiolitis, pneumonia, and lung cancer. Both the binary and multiclass classifiers had classifying accuracies of 100%.

Brattain et al. (2013) use Gaussian or statistical operations to either score or classify LUS. Gaussian operations are convenient because of their low computational weight. However, they do not share the same level of generality as NN’s and as such they are trained on narrower data sets and are prescribed in narrower conditions. In Brattain et al. (2013), a statistical B-line scoring system was developed. Depending on the severity of the B-lines presented, the images were given a score between 0 and 4 using angular features and thresholding. This method analyzed segmented features and determines the severity of the B-lines depending on five conditions: 1) Mean of a B-line column; 2) Column length above half-maximum; 3) Value of the last row of a column; 4) Ratio of the value of the last row over maximum for that column; and 5) Ratio of the value for the midsection of a column over maximum for that column. If these five features exceeded predefined thresholds, the image column is a B-line severity associated with it. However, as per Anantrasirichai et al. (2016) this method is not robust as it is prone to being greatly affected by noise and image intensity meaning the threshold values must be changed depending on the quality of the images being analyzed.

Table 1 provides a comparison of the accuracies of assorted classification methods found in literature.

**TABLE 1:** Comparison of LUS image classification methods.

Method	Author	Objective	Accuracy	Sensitivity	Specificity
Supervised feed forward ANN (2018)	Correa et al.	Pediatric Pneumonia	—	90.9%	100%
ANN (2016)	Barrientos et al.	Pneumonia	—	91.5%	100%
CNN (2020)	Born et al.	COVID-19	92%	96%	79%
CNN (2020)	Cristiana et al.	B-lines (presence)	94%	—	—
CNN (2020)	Cristiana et al.	B-line (severity)	54%	—	—
CNN (2019)	van Sloun and Demi	B-lines (*in-vitro*)	91.7%	91.5%	91.8%
CNN (2019)	van Sloun and Demi	B-lines (*in-vivo*)	89.2%	87.1%	93%
CNN (2018)	Kulhare et al.	Multiple Abnormalities	—	¿85%	¿85%
SVM Classifier (2020)	Carrer et al.	COVID-19	88–94%	—	—
RVM Classifier (binary) (2016)	Veeramani and Muthusamy	Healthy lung	100%	100%	100%
RVM Classifier (multiclass) (2016)	Veeramani and Muthusamy	Multiple Abnormalities	100%	100%	100%
Stochastic Method (2013)	Brattain et al.	B-lines	100%	—	—

## 5 Discussion

The previous sections discussed different methods to identify manifestations of COVID-19 in lung ultrasound images. Several challenges exist in order to implement these methods in a useful clinical setting that can effectively assist healthcare professionals during the course of the pandemic, autonomously identify manifestations of COVID-19 in LUS images, and assess the severity of the infection according to the grading scale proposed in Soldati et al. (2020). The most important practical considerations are related to the quality of the ultrasound images. This means that the system must guide healthcare professionals during LUS examinations and ensure appropriate image quality is obtained regardless of the operator’s experience and hardware, and the image processing method must be integrated into a portable ultrasound system.

### 5.1 Augmented LUS Images for Operator Guidance

Providing health care practitioners with an alternative to the time consuming and ionizing CT and X-ray scans would reduce the loading on the current medical system. However, the increasing need for lung imaging in hospitals, long-term care homes, and clinics, can lead to a shortage of sonographers. A reduction in that additional load can be sought in the form of a device to be used by personnel other than trained sonographers to either assist in triaging incoming patients or be used as a bedside monitoring tool.

The biggest challenge in LUS is that image segmentation and classification requires quality images. One possible way to assist the operation in this regard is to overlay processed images on top of the original LUS images. For example, one can consider presenting diagnostic information and a real time assessment of the image quality over the original image to intuitively guide the operator as in Moshavegh et al. (2016) and Moshavegh et al. (2018). Image overlay on top of the LUS image can indicate the current state of the image and how the operator can target specific features in the images. Further, following the recommendations outlined in the LUS-based diagnosis of COVID-19 standardization protocol proposed in Soldati et al. (2020), such a software may guide the operator to ensure that:

1. The focal point of the image is set on the pleural line. Using a single focal point and setting it at the right location has the benefit of optimizing the beam shape for sensing the lung surface. At the focus, the beam has the smallest width and is therefore set to best respond to the smallest details.
2. The mechanical index is kept below 0.7. Mechanical index is an indication of an ultrasonic pressure ability to cause micromechanical damage to the tissue. The mechanical index decreases as the focal zone moves further away from the transducer, hence it can become a concern given the previous point, in particular for a long observation time as it is required for LUS. The mechanical index can be changed with the frequency of the beam.
3. The image is not saturated. Saturation occurs when the signal strength of the echo signals is too high making the pressure/echo relationship no longer linear. This has the effect of distorting the signals images, giving rise to completely white areas in the image, which can be easily identified in the software. Control gain and mechanical index can be adjusted to prevent saturation.
4. The ultrasound probe is properly oriented to provide oblique scans. The image features needed in the image processing algorithm are clear.

### 5.2 Integration With a Portable Hand-Held Ultrasound

High frequency linear array probe is suggested to be used for minor subpleural lesions, as it can provide rich information and improve diagnostic accuracy. In the setting of COVID-19, experts suggest that wireless ultrasound transducers and tablets are the most appropriate ultrasound equipment for diagnosis, Soldati et al. (2020). These devices can easily be wrapped in single-use plastic covers, reducing the risk of viral contamination and making sterilization procedures easy. Furthermore, such devices can range between $4,000 and $8,000, which is a fraction of the cost of regular ultrasound machines. In cases of unavailability of these devices, portable machines dedicated to use for patients with COVID-19 can be still used, although more care for sterilization is necessary.

On a software front, the QLUSS and RVM classification methods presented in Section 4.2, respectively, seem well suited for a handheld solution. The QLUSS system has a low computational weight attached to it and is able to operate in real time, which is an asset for front line workers. The RVM method is capable of classifying which lung disease from a list of potentials is present in the LUS images and for processing of images off line. These methods also have the added benefit of requiring small databases, which could be stored in the handheld device itself or on a nearby computer. Using an off-line, i.e., a method which segments and classifies images after they are taken, solution is critical in certain parts of the world due to the possibility of data breach. Or online solutions—i.e., a solution that attempts to segment and classify images live as they are being taken—are simply not feasible due to lack of infrastructure. A portable hand-held United States device would require local storage which could be updated when new data was made available. An alternative option would be to access a database stored online via the Internet, as in Born et al. (2020a), if the infrastructure is available.

### 5.3 Probe Tracking

Probe tracking, a well documented and researched field Bouget et al. (2017), gives the sonographer the ability to see in real-time the position and orientation of the ultrasound probe. It can be done by integrating a motion sensor into the probe itself. By putting a position stamp on each United States frame would assist in identifying and mapping intercostal tissue and bones which may inadvertently cause black spots in the images, which are of no use. Further, the ability to know each United States images relative location to one another would allow the creation of 3D maps to assist in diagnosis.

## 6 Conclusion

Current advancements in ultrasound image processing provides health care practioners a means of imaging lungs to diagnose COVID-19. The methods presented in this article may aid in interpreting LUS images autonomously or semi-autonomously, thus allowing doctors without sonogoraphic training to diagnose COVID-19. Integration of image processing for COVID-19 diagnosis into handheld ultrasound machines can be used for beside monitoring, as a triaging tool for quickly diagnosing the severity of COVID-19 present.

As the COVID-19 pandemic and its characteristic traits are so new to medical research, there is a severe lacking of databases with significant resources. But as with every disease that has come before, those resources will come with time. Further, those databases combined with LUS will allow for more in-depth, greater diagnostic tools.

## Footnotes

1 A sequence of two morphological operations, opening and closing, that closes small gaps, see Sternberg (1986) for details.

2 Specificity is defined as true negatives/(true negatives + false positives), sensitivity is defined as true positive/(true positive + false negative), accuracy is defined as (true positive + true negative)/(all positives + all negatives).
